# Validation of a Novel Clinical Prediction Score for Severe Coronary Artery Diseases before Elective Coronary Angiography

**DOI:** 10.1371/journal.pone.0094493

**Published:** 2014-04-08

**Authors:** Zhang-Wei Chen, Ying-Hua Chen, Ju-Ying Qian, Jian-Ying Ma, Jun-Bo Ge

**Affiliations:** 1 Department of Cardiology, Zhongshan Hospital affiliated to Fudan University, Shanghai, China; 2 Department of Endocrinology Medicine, East Hospital affiliated to Tongji University, Shanghai, China; University Heart Center, Germany

## Abstract

**Objectives:**

Coronary artery disease (CAD) severity is associated with patient prognosis. However, few efficient scoring systems have been developed to screen severe CAD in patients with stable angina and suspected CAD before coronary angiography. Here, we present a novel scoring system for CAD severity before elective coronary angiography.

**Methods:**

Five hundred fifty-one patients with stable angina who were admitted for coronary angiography were enrolled in this study. Patients were divided into training (n = 347) and validation (n = 204) cohorts. Severe CAD was defined as having a Gensini score of 20 or more. All patients underwent echocardiography (ECG) to detect ejection fraction and aortic valve calcification (AVC). Multivariable analysis was applied to determine independent risk factors and develop the scoring system.

**Results:**

In the training cohort, age, male sex, AVC, abnormal ECG, diabetes, hyperlipidemia, high-density lipoprotein cholesterol, and low-density lipoprotein cholesterol were identified as independent factors for severe CAD by multivariable analysis, and the Severe Prediction Scoring (SPS) system was developed. C-indices of receiver operating characteristic (ROC) curves for severe CAD were 0.744 and 0.710 in the training and validation groups, respectively. The SPS system also performed well during calibration, as demonstrated by Hosmer-Lemeshow analysis in the validation group. Compared with the Diamond-Forrester score, the SPS system performed better for severe CAD prediction before elective coronary angiography.

**Conclusions:**

Severe CAD prediction was achieved by analyzing age, sex, AVC, ECG, diabetes status, and lipid levels. Angina patients who achieve high scores using this predicting system should undergo early coronary angiography.

## Introduction

Coronary artery disease (CAD) is a leading cause of morbidity and mortality worldwide, and the costs of invasive and noninvasive diagnostic methods, which are performed to identify the presence and severity of CAD, are increasing dramatically. The severity of CAD, which can be analyzed by Gensini score [1] or Syntax score [2], has been shown to be associated with short- and long-term cardiovascular risk [3]. Both of these scoring systems consider coronary anatomy, artery morphology, and severity of stenosis in lesions [4]. Therefore, in order to identify severe CAD (defined as ≥70% stenosis in the proximal left anterior descending artery, equal to a Gensini score ≥20 points) and help cardiologists make treatment decisions, several clinical risk models have been developed. These models, including the EuroHeart score, Clinical SYNTAX score, Mayo clinic risk score, etc., predict long-term cardiovascular risk by analyzing clinical risk factors and severity of CAD [2], [5]–[7]. However, all of these models require the results of coronary angiography or coronary computed tomography angiography. Because many patients with atypical chest pains or even asymptomatic patients may be at risk of severe CAD, it is not feasible to suggest that every patient undergo coronary angiography in this setting. Several clinical scoring systems, such as Diamond-Forrester score [8], Framingham risk score (FRS), and systematic coronary risk evaluation (SCORE) have been established to assess CAD risk by analyzing classical risk factors, such as chest pain type, age, gender, blood pressure, smoking status, diabetes status, and cholesterol level [9]–[10]. However, the effectiveness of these models for prediction of severe CAD by clinical risk factors is not sufficient. While the Diamond-Forrester score has been recommended to predict CAD before coronary angiography, its performance in severe CAD prediction is unknown. Therefore, it is necessary to develop a highly efficient and convenient screening model for severe CAD without requiring coronary angiography results.

The aim of this study was to develop a novel risk scoring system to guide early invasive coronary angiography in angina patients using analysis of clinical risk factors, electrocardiography (ECG), and echocardiography. Additionally, we attempted to demonstrate whether this risk scoring system was effective to predict severe CAD before coronary angiography, and we compared the performance of this system with that of the Diamond-Forrester score for prediction of CAD and severe CAD. Further analyses were also carried out to detect the association between scores from our novel system and Gensini scores.

## Methods

### 1. Study Population and Grouping

From October 2011 to September 2012, a total of 551 angina patients referred to our hospital for elective coronary angiography were enrolled in this study. The inclusion criteria were as follows: (1) 18 to 85 years of age; (2) providing a complete clinical history; and (3) normal preprocedural troponin T (below the 10% coefficient of variation [CV] value, <0.03 ng/mL [11]) and creatine kinase (CK)-MB (<23 U/L). The exclusion criteria were as follows: (1) underwent coronary angiography or computerized tomography angiography previously; (2) diagnosed with acute coronary syndrome; (3) evidence of elevated cardiac troponin T (≥0.03 ng/mL) or CK-MB (≥23 U/L) before coronary intervention; (4) presence of heart failure, diagnosed by clinical presentation, echocardiography (ejection fraction [EF] <40%), and N-terminal-pro-brain natriuretic peptide (>300 pg/mL); (5) presence of cardiomyopathy, congenital heart disease, or heart valve disease; (6) underwent recent surgery or trauma; (7) presence of active chronic inflammation, renal failure, dysfunction of hematological and immunological systems, carcinoma, or a condition treated with immunosuppressive agents.

From this patient population, 347 patients (continuously enrolled by hospitalization time) were enrolled into the training cohort for risk factor identification and predicting system development, while the other 204 patients were enrolled for validation of the prediction system. First, the training cohort was subjected to univariate and multivariate analyses to identify independent risk factors for severe CAD (defined in section 2.6). Each prediction score was evaluated by determining the odds ratio (OR) of each risk factor in multivariate analysis, and the total prediction score for severe CAD was calculated by summating all prediction scores. In order to demonstrate the clinical practicality and efficacy of this new prediction score for severe CAD screening, receiver operating characteristic (ROC) curves and Hosmer-Lemeshow analysis were analyzed in the validation cohort to examine model performance in terms of discrimination and calibration.

### 2. Ethics Statement

We provided a written informed consent form to participants in our study and explained the entire study procedure to each patient. This study and consent procedure were approved by our local ethics committee (Ethics Committee of Zhongshan Hospital affiliated to Fudan University), and were carried out in accordance with the principles of the Declaration of Helsinki.

### 3. Medical History Records and Laboratory Measurements

The clinical characteristics of all patients, including gender, age, and previous histories of smoking, hypertension, diabetes, and hyperlipidemia, were recorded before coronary angiography. Fasting blood samples were collected before angiography to detect blood biochemistry, and complete blood cell counts was performed. All biochemical parameters, including total cholesterol, total triglycerides, low-density lipoprotein cholesterol (LDL-C), high-density lipoprotein cholesterol (HDL-C), apolipoprotein-A, apolipoprotein-B, uric acid, and creatinine concentrations, were analyzed.

Patients were defined as hypertensive (JNC VII Guidelines), if they had a systolic pressure greater than 140 mmHg or a diastolic pressure greater than 90 mmHg, or if they were being treated with an antihypertensive medication. Patients were considered to have type II diabetes mellitus (DM) if they were previously diagnosed or following the 2010 American Diabetes Association (ADA) diabetes diagnostic criteria. The definition of hyperlipidemia was taken from the NHLBI ATP III prevention guidelines.

### 4. Electrocardiography and Echocardiography

Admission ECG was performed for each patient. Abnormal ECG was defined as having Q waves in multiple leads, ST-T-wave inversions, left/right bundle-branch blockage, or left ventricular hypertrophy [12]. Echocardiography was performed in all patients using a Philips IE33 instrument (Philips, Netherlands) with a 2–3.5 MHz transducer (X3-1), and left ventricular EF and aortic valve calcification (AVC) were detected. Two-dimensional assessment of the aortic valve was performed on the basis of the parasternal long-axis and short-axis views, where abnormalities of the aortic valve were coded as representing AVC, aortic stenosis, a bicuspid aortic valve, or aortic-valve regurgitation. AVC was defined as focal areas of increased echogenicity and thickening of the aortic valve leaflets without restriction of leaflet motion on transthoracic echocardiography [13]–[14]. Observers who made the diagnosis of AVC were blinded to the results of coronary angiography.

### 5. Coronary Angiography and Severity CAD Identification

Elective coronary angiography was performed in all patients after admission. A patient was considered to have CAD when a stenosed lesion resulting in a 50% or greater reduction in lumen diameter existed in at least one of the coronary arteries. Furthermore, the total number of stenosed vessels was represented as the number of major stenoses in epicardial arteries with at least one stenosed lesion (≥50% reduction of lumen diameter), including the left anterior descending artery (LAD), left circumflex artery (LCX), right coronary artery (RCA), and left main artery (LM).

The severity of CAD was evaluated by Gensini score [1]. Gensini score grades narrowing of the lumen as follows: 1, 1%–25% occlusion; 2, 26%–50% occlusion; 4, 51%–75% occlusion; 8, 76%–90% occlusion; 16, 91%–99% occlusion; and 32, total occlusion. This score is multiplied by a factor accounting for the importance of the lesion position in the coronary arterial tree, such as 5 for LM, 2.5 for proximal LAD, and 1 for proximal RCA. The severity of the disease is expressed as the sum of the scores for individual lesions. Gensini score and number of stenosed vessels were recorded by observers who were blinded to the results of laboratory testing and study grouping.

Patients with Gensini scores of 20 or more were defined as having severe CAD, which was approximately equal to one stenosed lesion of 70% or more in the proximal left anterior descending artery.

### 6. Calculation of the Diamond-Forrester Score

First, we classified angina type as typical, atypical, or nonspecific [15]. Typical chest pain was defined as having (i) substernal chest pain or discomfort; (ii) pain provoked by exertion or emotional stress; and (iii) pain relieved by rest and/or nitroglycerine. Atypical chest pain was defined as having 2 of the above-mentioned criteria. If one or none of the criteria was present, the patient was classified as having nonspecific chest pain. The Diamond Forrester model was developed to calculate the probability of CAD and considers age (only patients 30 to 70 years old were considered for this model), sex, and type of chest pain [8].

### 7. Statistical Analysis

All statistical analyses were performed with SPSS software 19.0. Data were presented as the percentage or mean ± standard deviation (SD). The database was randomly divided into 2 parts. The first part was used to develop the scoring system (i.e., the training cohort). The second part was used to validate the scoring system (i.e., the validation cohort). In the training cohort, chi-squared tests were used to compare the frequencies of categorical variables, and Student’s *t* or correction *t* tests were used to compare means for continuous variables. Multivariate analysis (logistic) was performed to identify independent risk factors for severe CAD patients (variables with a significance level of *P*<0.05 in a univariate test were used in multivariate analysis). According to each OR of independent variables, we set each prediction score and calculated the total prediction score. Then, a prediction risk curve of severe CAD was established. The area under the ROC curve was used to test the discriminatory capability of the total prediction score, and Youden’s index was applied to establish the optimal cutoff. ROC and Hosmer-Lemeshow analyses were also applied in the validation cohort to demonstrate the goodness of fit for the novel prediction scoring system. Correlation analysis (Spearman test) was performed to evaluate correlations between this prediction score and the Gensini score. All *P*-values were 2-sided, and *P*<0.05 was considered to indicate statistical significance.

## Results

### 1. Population Baseline Characteristics

A total of 551 patients with exertional chest tightness or chest pain were enrolled in this study for selective coronary angiography. There were 379 men (average age, 62.7±9.8 years) and 172 women (average age, 66.2±8.9 years). The prevalences of hypertension, diabetes, hyperlipidemia, and AVC were 70.8% (390 patients), 30.9% (170 patients), 30.7% (169 patients), and 34.3% (189 patients), respectively. A total of 440 (79.8%) patients were diagnosed as having CAD by coronary angiography.

In the training cohort (n = 347), there were 202 patients diagnosed with severe CAD by Gensini score (≥20), and the average Gensini score was significantly higher in the severe CAD group than in the mild/moderate CAD group (46.3±28.0 vs. 7.1±6.0, respectively, *P*<0.01). The baseline characteristics of patients with or without severe CAD are shown in Table 1. Compared to patients with nonsevere CAD, patients with severe CAD were older (65.1 vs. 62.4 years, *P*<0.01) and had higher prevalences of hyperlipidemia (39.6% vs. 23.4%, *P*<0.01), diabetes (37.6% vs. 21.4%, *P*<0.01), and AVC (42.1% vs. 30.6%, *P*<0.01).

**Table 1:** Demographic data of patients with and without severe coronary artery disease.

	Severe CAD (n = 202)	Non-severe CAD (n = 145)	*P*
**Clinical factors**:			
Male (%)	149 (73.8%)	89 (61.4%)	**0.014**
Age (year)	65.1±9.6	62.4±8.9	**<0.01**
Hypertension (%)	144 (71.3%)	97 (66.9%)	0.381
Hyperlipidemia (%)	80 (39.6%)	34 (23.4%)	**<0.01**
Diabetes (%)	76 (37.6%)	31 (21.4%)	**<0.01**
Smoking (%)	92 (45.5%)	60 (41.4%)	0.441
**Laboratory test (admission)**:			
Serum Creatinine (μmol/L)	78.9±18.8	73.7±17.2	**<0.01**
Serum Uric acid (mmol/L)	352.2±88.1	342.1±86.2	0.287
Fibrinogen (g/L)	293.4±59.7	275.1±58.2	**<0.01**
Total cholesterol (mmol/L)	4.33±1.27	4.11±0.88	0.055
Triglyceride (mmol/L)	1.91±1.35	1.79±1.29	0.409
LDL-C (mmol/L)	2.45±1.11	2.16±0.69	**<0.01**
HDL-C (mmol/L)	1.08±0.25	1.19±0.29	**<0.01**
Lipoprotein-a (mmol/L)	208.4±186.7	175.4±170.5	0.089
Apo-A (mmol/L)	1.09±0.22	1.09±0.24	0.878
Apo-B (mmol/L)	0.86±0.23	0.76±0.22	**<0.01**
**Electrocardiography**			
Abnormal ECG (%)	117 (57.9%)	52 (35.9%)	**<0.01**
**Echocardiography**			
Ejection fraction (%)	62.4	64.5	0.465
AVC	85 (42.1%)	34 (30.6%)	**<0.01**
**Coronary angiography**:			
Gensini score	46.3±28.0	7.1±6.0	**<0.01**
Number of stenosed vessels	2.26±0.88	0.6±0.6	**<0.01**

AVC: aortic valve calcification; CAD: coronary artery disease; ECG: electrocardiography;

HDL-C: high density lipoprotein cholesterol; LDL: low density lipoprotein cholesterol;

### 2. Identification of Determinants of Severe CAD by Multivariate Logistic Regression

In the training cohort, multivariate logistic regression analysis was used to evaluate the association between severe CAD and risk factors. In this analysis, severe CAD was employed as a dependent variable, while age, male sex, AVC, abnormal ECG, diabetes, hyperlipidemia, HDL-C, LDL-C, and serum creatinine were set as independent variables (Table 2). After adjustment for other associated factors, we found that the risk of severe CAD was increased independently in patients who were male or had AVC, abnormal ECG, diabetes, hyperlipidemia, or abnormal LDL-C. Serum levels of HDL-C were associated with a 69% risk reduction for severe CAD (OR = 0.310, 95% confidence interval [CI] = 0.116–0.827, *P = *0.019).

**Table 2:** Multivariate logistic regression analysis for severe CAD in patients with chest pain.

	*r*	95% confidence intervals	*P*
AVC	2.580	1.538–4.329	**<0.01**
Abnormal ECG	2.148	1.333–3.460	**<0.01**
diabetes	1.966	1.149–3.365	**0.014**
Male	1.880	1.095–3.226	**0.022**
Hyperlipidemia	1.794	1.054–3.053	**0.031**
LDL-C	1.516	1.136–2.024	**<0.01**
HDL-C	0.310	0.116–0.827	**0.019**

AVC: aortic valve calcification; CAD: coronary artery disease; ECG: electrocardiography;

HDL-C: high density lipoprotein cholesterol; LDL: low density lipoprotein cholesterol;

### 3. New Prediction Scoring System and Risk Curves for Severe CAD

**Figure 1:**
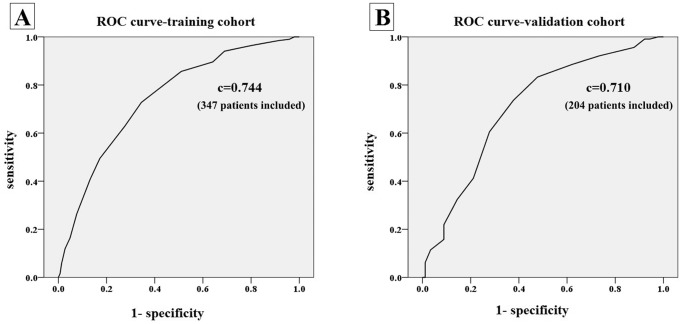
Receiver operating characteristic (ROC) curve in training (A) and validation (B) cohort. (347 and 204 patients were analyzed in training and validation cohorts respectively).

According to each OR of independent variables in multivariate logistic regression analysis, we set the prediction score of each risk factor (Table 3). The total prediction score was determined by summating the 7 individual prediction scores. Then, ROC curve analysis was applied to detect the efficacy of the new prediction score, and the area under the ROC curve (AUC) was analyzed. Using the 7 defined factors, the AUC was 0.734 (*P*<0.01). After adding age (>65 years), the AUC was increased to 0.744 (*P*<0.01), as shown in Figure 1-A. Therefore, age was added as a risk factor for predicting severe CAD.

**Table 3:** Scoring system predicting for severe CAD in patients with chest pain.

Risk factor	Range	Single score
AVC	yes	**3**
Abnormal ECG	yes	**3**
Diabetes	yes	**2**
Male	yes	**2**
Hyperlipidemia	yes	**2**
LDL-C (mmol/L)	<1.8	**0**
	1.8–2.2	**1**
	≥2.2	**2**
HDL-C (mmol/L)	≥1.2	**0**
	1.0–1.2	**1**
	<1.0	**2**
Age (years)	<65	**0**
	≥65	**2**
**Severe Predicting Score**		**18**

**Figure 2:**
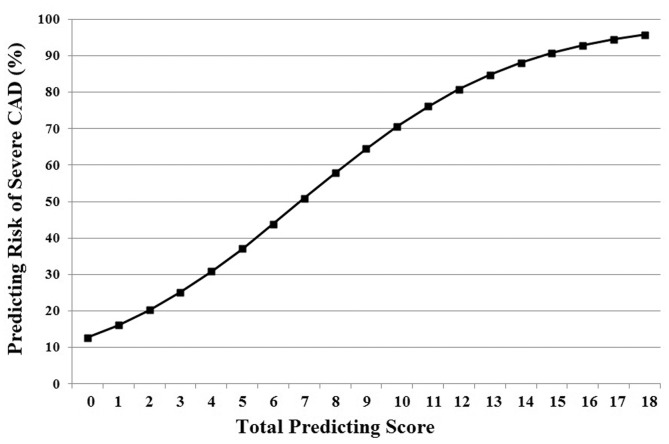
Predicting risk of severe coronary artery disease by this scoring model.

AVC: aortic valve calcification; CAD: coronary artery disease; ECG: electrocardiography;

HDL-C: high density lipoprotein cholesterol; LDL: low density lipoprotein cholesterol;

In order to calculate the prediction risk score for severe CAD, we set severe CAD as a dependent variable and set the new total prediction score as an independent variable in multivariate analysis. Then, “Y = constant+βX” was obtained from logistic analysis, and each prediction risk score for severe CAD was calculated. Figure 2 demonstrates the prediction risk score curve for severe CAD according to each score. For example, an angina patient with a new score of 3 points had less than 25% risk of severe CAD, and this risk increased to 60% when the score was 9 points. The optimal cutoff for prediction of severe CAD was 8 points, with a sensitivity of 72.8% and a specificity of 65.5%.

### 4. Validation Test in the Validation Cohort

In order to demonstrate the clinical practicality and efficacy of this new prediction score for severe CAD screening, the scoring system and prediction risk curves were retested in the validation cohort. First, the total prediction score was calculated according to Table 3. Then, an ROC curve was established. The AUC of the ROC in the validation cohort was 0.710 (*P*<0.01, Figure 1B). Hosmer-Lemeshow analysis was also performed in the validation cohort to evaluate the goodness of fit for the prediction score. The Hosmer-Lemeshow *P*-value was 0.425 (>0.05), which indicated that the prediction risk curve was effective for severe CAD screening.

We also investigated the model performance in different subgroups, as shown in Table 4. The results demonstrated that this scoring system had similar performance in older, hypertensive, and diabetic patients. However, it performed less well in male (0.690 vs. 0.778 in female) patients and patients with kidney dysfunction (0.639 vs. 0.774 in patients with normal kidney function).

**Table 4:** Subgroup analysis for the scoring system in the whole cohort.

Risk factor	Subgroup	Severe CAD/Sample	C-index	H-L p-value
**Gender**	Male	236/379	**0.690** *	0.308
	Female	80/172	**0.778** *	0.477
**Age (year)**	≥65	165/259	0.706*	0.291
	<65	151/292	0.734*	0.459
**Hypertension**	Hypertension	235/390	0.719*	0.390
	Non-hypertension	81/161	0.749*	0.848
**Diabetes**	Diabetes	117/170	0.715*	0.770
	Non-diabetes	199/381	0.718*	0.288
**Serum creatinine (μmol/L)**	≥80	136/214	**0.639** *	0.747
	<80	180/337	**0.774** *	0.710

*means the p-value <0.05; CAD: coronary artery disease; H–L: Hosmer–Lemeshow;

### 5. Extent of CAD and the New Scoring System

Patients were also classified into 4 groups by different Severe Prediction Scoring (SPS) results in the whole cohort: A, low-risk group, 0–3 points; B, intermediate-risk group, 4–7 points; C, high-risk group, 8–11 points; and D, very-high-risk group, more than 12 points. In higher scoring groups, the prevalences of CAD, severe CAD, and multivessel stenosis were increased significantly, as shown in Table 5. The association between SPS and Gensini scores was also analyzed in the whole cohort. We found that SPS scores were positively associated with Gensini scores for each patient (*r = *0.414, *P*<0.01).

**Table 5:** Prevalence and severity of CAD in different novel scoring groups.

	A: 0–3 points (n = 51)	B: 4–7 points (n = 185)	C: 8–11 points (n = 218)	D: >11 points (n = 97)	*P*
**CAD (%)**	30 (58.8%)	130 (70.3%)	190 (87.2%)	90 (92.8%)	**<0.01**
**Severe CAD patients (%)**	12 (23.5%)	73 (39.5%)	153 (70.2%)	78 (80.4%)	**<0.01**
**Gensini score**	14.1±19.3	20.3±22.3	33.6±28.1	43.9±33.6	**<0.01**
**Stenosed vessels**	0.9±0.9	1.2±1.0	1.8±1.1	2.2±1.0	**<0.01**

CAD: coronary artery disease;

### 6. Compare with Diamond-Forrester Scores for Predicting Performance

**Figure 3:**
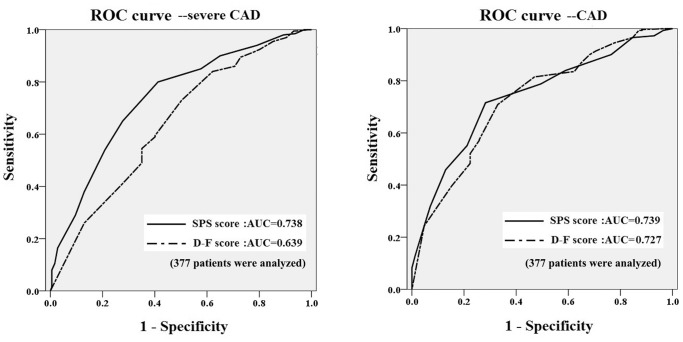
Compared receiver operating characteristic (ROC) curve between our SPS system and Diamond-Forrester score in severe CAD and CAD predicting. (377 patients were analyzed).

As a classic scoring system for CAD prediction, the Diamond-Forrester score was calculated in patient ages 30–70 years (only 377 patients met this age condition for Diamond-Forrester score calculation). The average Diamond-Forrester score was 68.3±27.3 in these 377 patients. After dividing patients according to the type of chest pain, we found that patients with typical chest pain had significant associations between the prevalence of CAD and the SPS score, as shown in Table 6. As compare to the Diamond-Forrester score, our SPS system seemed to perform better for prediction of severe CAD (C-index, 0.738 vs. 0.639, respectively), while they had similar performance for prediction of nonsevere CAD (C-index, 0.739 vs. 0.727, respectively), shown in Figure 3.

**Table 6:** Prevalence of CAD and risk score in different types of chest pain (377 patients met 30–70 years condition for Diamond–Forrester score).

	Typical angina n = 190	Atypical angina n = 132	Non-specific CP n = 55	*P*
**CAD (%)**	168 (88.4%)	95 (72.0%)	29 (52.7%)	**<0.01**
**Age (years)**	59.9±6.6	58.5±6.6	55.2±7.9	**<0.01**
**Male (%)**	33 (60.0%)	99 (75.0%)	141 (74.2%)	0.106
**D-F score**	91.0±4.6	56.9±11.1	17.9±6.4	**<0.01**
**SPS score**	8.1±3.2	7.1±3.4	5.9±3.0	**<0.01**

CAD: coronary artery disease; CP: chest pain; D–F score: Diamond–Forrester score;

SPS score: Severe Predicting Score.

## Discussion

As we have discussed, several clinical scoring systems, including the Diamond-Forrester score [8], FRS [9], and SCORE [10], have been established to assess CAD risk by classical risk factors; however, the usefulness of these systems in predicting severe CAD before coronary angiography has not been established. In our study, we developed a novel risk scoring system for severe CAD screening by clinical risk factors, ECG, and echocardiography before coronary anatomic analysis. ROC and Hosmer-Lemeshow analysis in both the training and validation cohorts demonstrated that this model had good performance in terms of discrimination (C-indices of 0.744 and 0.710, respectively) and calibration (Hosmer–Lemeshow *P*-values of 0.824 and 0.425, respectively). Therefore, our results suggested that this system could help cardiologists identify severe CAD in a great number of angina patients before coronary angiography, thereby indicating whether early invasive coronary angiography should be performed. Especially in the setting where catheter lab resources are scant and waiting time for the examination is long, this score system could screen sever CAD patients by higher score conveniently. As low and intermediate score cohort, other non-invasive examinations and further follow-up could be considered.

Distinguished from conventional scoring systems for CAD risk prediction or prognosis in patients with CAD [3], [5], [9], our scoring system employed AVC, an echocardiography characteristic, for evaluation of CAD severity. As a characteristic of aging, AVC is also considered a frequent cause of aortic stenosis [16]. However, recent data have challenged this concept, showing that AVC is an active and highly regulated process, with histological similarities to atherosclerosis [14], [17]–[18]. In our previous cross-sectional study [19], [20], we found that AVC was still associated with CAD and CAD severity even after adjustment for aging itself. However, few other studies have considered using AVC for evaluation of CAD or CAD severity. As demonstrated by the multivariate analysis in this study, AVC increased the risk of severe CAD by 158% (Table 2), which demonstrated its independent influence on severe CAD. More importantly, the ROC of our scoring system was reduced to 0.687 in the validation cohort if AVC was excluded. According to these findings, AVC could be helpful for screening of severe CAD in combination analysis.

As compared with the classic scoring system, i.e., the Diamond-Forrester score [8], which is generally applied for predicting CAD risk, our SPS system exhibited similar performance in CAD prediction (C-indices of 0.739 and 0.727, respectively). However, our SPS system was significantly superior to the Diamond-Forrester score for prediction of severe CAD (C-indices of 0.738 vs. 0.639, respectively, shown in Figure 3). This implied that the SPS system was effective for predicting CAD and severe CAD before elective coronary angiography. Moreover, subgroup analysis by different variants, such as age, gender, hypertension, diabetes, and creatinine level, demonstrated the good performance of the SPS system in terms of discrimination and calibration. However, it also revealed that our model was less useful for prediction of severe CAD in patients with relatively higher serum creatinine (0.639 vs. 0.774 in patients with normal kidney function). Early kidney injury may influence the metabolism of lipids and glucose, resulting in poorer performance of our scoring system.

Another interesting point was that our scoring system (which was determined by clinical factors) exhibited a significant positive relationship with Gensini score, which was obtained from the result of invasive coronary angiography examination. Gensini score involves analysis of both coronary artery morphology and percentage of stenosis [1], which is associated with long-term cardiovascular outcomes [3]. Therefore, these data partly indicated that our novel scoring system, using clinical risk factors, may also have the potential to predict the cardiac prognosis. However, further random, prospective clinical studies are needed to document this association.

This study also had several limitations. First, the study population was relatively small, and the study design was cross-sectional, with a lack of follow-up data. Additionally, echocardiography cannot distinguish calcification from fibrosis of the aortic valve; therefore, severe fibrosis may have been misdiagnosed as calcification. Finally, the definition of severe CAD was set according to the results of angiography and Gensini scores. Thus, this system could not explain the association between the occurrence of myocardial ischemia and stenosis. Further prospective studies are needed to clarify the efficacy of our scoring system.

## Conclusions

Severe CAD prediction in patients with stable angina was achieved using our novel scoring system, which combined clinical risk factors and echocardiography. This scoring system performed well in patients prior to coronary angiography, which could be helpful for making diagnostic decisions.
